# Masculine men do not like feminine wording: The effectiveness of gendered wording in health promotion leaflets in the UK

**DOI:** 10.1371/journal.pone.0273927

**Published:** 2022-10-27

**Authors:** Katherine Baxter, Barbara Czarnecka, Bruno Schivinski, Maria Rita Massaro

**Affiliations:** 1 Department of Business Management and Marketing, Liverpool Hope University, Liverpool, United Kingdom; 2 Division of Management, Marketing and People, London South Bank University, London, United Kingdom; 3 School of Media and Communication, RMIT University, Melbourne, Australia; 4 Independent Researcher, Grosseto, Italy; University College London, UNITED KINGDOM

## Abstract

Following mixed-methods sequential design and drawing on the message-audience congruence concept and homophily theory, across three studies in the UK, we examined the effect of gendered wording and endorser’s gender on the effectiveness of leaflets promoting walking. In Study 1, a mall-intercept study achieved 247 completed questionnaires. Results demonstrated that men and women indicated the highest behavioural intentions for communal wording presented by a male endorser. However, pairwise comparisons revealed that when the wording of the advert was agentic and the endorser was male, males indicated significantly higher scores of behavioural intentions compared with females. Attitude towards the ad for women was highest for communal wording/female endorser; for men it was for agentic wording/male endorser. In Study 2, consumers’ views towards the gendered content were explored in 20 semi-structured interviews. In study 3 we examined the impact of the respondent’s gender role identity on gendered content effectiveness. Overall, when controlled for level of gender role identity, only masculine males evaluated leaflets featuring communal wording negatively which suggests that wording matters only for masculine males, but not for other men and women. Theoretically, we identified that gender-based message-respondent congruence is not a necessary aspect of communications to be effective, except for one group: masculine males. Our study identified dominant gender role identity as a factor that explained respondents’ preferences for presented stimuli. Specifically, males who display masculine gender role identity differ in evaluations of communal wording from all other groups. Social and commercial marketers who target men and women with exercise-related services should consider the use of agentic wording endorsed by a male endorser when targeting masculine men to increase the likelihood of eliciting positive attitudes towards the communication. However, such distinctions should not be associated with differences in women’s evaluations or men who do not report masculine gender role identity.

## Introduction

Gender effects have been examined in a wide range of contexts including communication [1–10]. Past studies demonstrated that gendered content of advertisements, including wording and endorser’s gender, may often influence responses to communication among men and women in a different way [11–14]. Whilst the impact of communication on behaviour is disputed, policy makers, practitioners and advertisers continue using communication to raise awareness and influence behaviours. This is especially so in instances where promoted behaviours, such as physical exercise, cannot be legislated for or regulated in other ways. In the UK, the government has introduced a range of social change communication initiatives to encourage behaviour change in this area [15,16], as well as established Office for Health Communication to focus specifically on raising the profile of health-related issues [17]. Often, such campaigns are targeted at specific genders. For example *This Girl Can* campaign in the UK is targeted specifically at women, who continuously remain less active than men [18].

Solving such public health problems is complex, and many aspects of communications strategies have been examined in the past [19–21]. We decided to look at how one approach to communication, that is using gendered content (i.e., masculine- and feminine-themed words, referred to as gendered wording, and presented by either male or female endorsers), affects individuals’ responses to the message. As such, this study focuses on examining persuasion effects of gendered content in health communication in the UK. Our motivation to focus on this question is driven by a substantial volume of research on the effectiveness of gendered content in contexts outside of health communication and outside the UK [13,22–26]. Hence little is known about the effectiveness of gendered content in health communication in the UK specifically. If gendered wording and respondent’s gender are such impactful variables in context of job adverts in Germany or Denmark [25,27], or branding efforts [28], could this message strategy be effective in health communication in the UK too? In addition, in the light of changing gender role expectations, it would be important to examine the effectiveness of gendered content at times of substantial evolution of gender roles and gender role expectations in the society [29].

Gendered wording is described as the use of words stereotypically associated with males or females. Two types of gendered wording are often discussed: agentic (using words stereotypically associated with males and relating to behaviours stereotypically associated with males), and communal (using words stereotypically associated with females and relating to behaviours stereotypically associated with females). For instance, words such as competitive, dominant, assertive or leader are associated with male stereotypes, while words such as support, understand, cooperate and interpersonal are associated with female stereotypes [26]. In addition to the wording, researchers found that who endorses the message also influences effectiveness [28,30–32]. Among the many characteristics of message endorsers is gender which has been examined in a wide range of contexts and has often been found to relate to message effectiveness [33,34].

Whilst endorser’s gender and gender role portrayal have been investigated in numerous advertising contexts [e.g., 35–37], gendered wording has only been researched predominantly in the context of job ads [12,27]. Moreover, no studies so far examined gendered wording effectiveness in the context of the UK. Hence, the aim of this paper is to examine the effect of gendered wording and endorser’s gender in exercise-related leaflets on individuals’ appraisals of those leaflets in the UK. Drawing on homophily theory [38], the message-audience congruency principle [39,40] and past research [41], we propose that men should respond more positively to agentic wording presented by a male endorser and women should respond more positively to communal wording presented by a female endorser.

Subsequently, via three studies, we examined attitude towards ad, and behavioural intention in a 2x2x2 survey experiment (Study 1) and explored consumers’ perceptions of gendered content in 20 semi-structured interviews (Study 2). In Study 3, we examined how the wording of the leaflet and the gender of the endorser interact with the recipient’s self-reported dominant gender role identity by measuring attitude towards ad, behavioural intentions and advert credibility.

Below, we present an overview of the existing literature on gendered content in marketing and health communications research. Subsequently, data analysis and results of the three studies are presented. Finally, we address the theoretical and practical implications of this study and present its limitations and suggestions for future research.

## Literature review and theoretical background

### Gendered wording and endorser’s gender

Gendered wording is defined as the use of “masculine- and feminine-themed words, such as those associated with gender stereotypes” [p.1, 27]. Agentic wording such as the words independent, assertive, ambitious, and decisive are stereotypically men-directed. Communal words such as warm, compassionate, sensitive, emotional are associated more with women [27,42–44]. Such gendered words are derived from gender stereotypes which translate into social role expectations and those expectations are often expressed in the way language is used when talking about men versus women [45–47].

Ample academic evidence suggests that despite marked changes in the way men and women are expected to behave, many people still believe agentic characteristics are more appropriate for and characteristic of men than for women (e.g., assertiveness, dominance, independence, brilliance), while communal characteristics are more noticeable in women than men (e.g., concern for others, kindness, emotional sensitivity) [48–50]. Those biases are also visible in the use of new technologies such as algorithms where search results for seemingly ‘neutral’ phrases show gender bias [51]. Social media users were also found to differ in their use of language, depending on the gender of the user [3]. Women were found to use certain phrases more often than men, and men were found to use certain phrases that women did not use.

Hentschel et al. [27] examined how female students, and older employees in Germany reacted to gender-stereotypical wording in job advertisements and found that older female employees perceived themselves as not belonging to the advertised jobs when wording was agentic regardless of gender of endorser. Younger women disliked genetic wording presented by a male, but did not respond negatively to the same wording presented by a female. Askehave and Zethsen [24] analysed Danish job advertisements for top executives to explore the use of gendered wording, and to examine individuals’ responses to the advertisements. The results demonstrated that the analysed job advertisements featured mostly agentic wording, and respondents assigned stereotypical male characteristics to the potential applicants for those advertised jobs. Wille and Derous [25] examined Belgian women’s responses to job adverts which featured descriptions of job requirements worded in a masculine and feminine manner. Gaucher, Friesen and Kay [26] examined how men and women perceived their suitability for an advertised job when the wording was communal versus agentic. For both studies, the results demonstrated that women were less likely to believe they belonged in a particular job when the advertisement used masculine wording and they rated masculine jobs as less appealing.

Elsewhere, agentic language was found to be effective in motivating a range of climate change-related behavioural intentions [52]. Bušljeta Banks, Dens, & De Pelsmacker [14] examined how men and women respond to the use of words to describe probability markers in ads and found that men had more pronounced responses to the presented words than women. Overall, these findings suggest inconclusive and different gender responses to wording in a number of contexts.

In addition to what is being said, who delivers the message has also been found to be important in the domain of message effectiveness. The effects of the endorser’s gender have been investigated in a wide range of contexts, leading to inconclusive outcomes [e.g., 53,54]. Recently, Hentschel et al. [27] found that younger German women responded to agentic wording in a positive way when it was presented by a female endorser but in a negative way when it was presented by a male endorser. Men, however, did not differentiate between wording or endorser’s gender. Such studies suggest the gender of endorser may change the way gendered wording is perceived, but published research is inconclusive. Moreover, as socio-cultural contexts change, and with them gender role expectations, individuals may respond to gendered content differently to what studies reported even a few years ago.

### Message-audience congruence and homophily theory

The message-audience congruence principle (often referred to as the ‘match-up’ hypothesis, or ‘fit’) rests on the proposition that messages similar to the characteristics of the audience should be more effective [28,55–58]. Amongst the different types of congruence (e.g., cultural, conceptual, perceptual), we draw on the construct of conceptual congruence to explain the relations between the gendered content of the leaflets, the respondent’s gender and dominant gender identity and message effectiveness. Conceptual congruence is a defined as “relatedness of conceptual attributes” [59]. Such c*ongruence* is the extent to which graphic and copy aspects of a message reflect a common theme [60,61]. Congruence also refers to how the content of the message ‘fits with’ the characteristics of a respondent. The proposition that congruent messages should be more effective draws on the congruity theory which posits that individuals usually identify with what is similar to their existing beliefs and values [62,63]. The similarity between the message and audience can rest on many characteristics (of the message and the audience) including gender identity as proposed by the homophily theory. Homophily theory posits that individuals tend to build networks with other individuals who have similar characteristics, such as gender (Lazarsfeld and Merton (1954)[cited in 38].

Congruence between the message content and the audience has been shown in many studies to increase the effectiveness of the messages because people tend to choose what is similar to them [64–67]. For example, Guan and So [68] investigated the effect of congruence between temporal message frames and respondent’s time orientation, and found that congruence was associated with more positive evaluations of those messages. In a different context, Godinho et al. [40] examined the effectiveness of loss and gain message frames to promote fruit and vegetable consumption and found that frames congruent with respondent’s motivational orientation were more effective than those which were incongruent. Similarly, Uskul, Sherman [69] researched how individuals respond to health communication messages and found support for the congruency effect: that is, culturally congruent messages were more effective in persuading respondents to have more positive attitudes and behavioral intentions towards the promoted health behavior. De Droog, Buijzen, & Valkenburg examined the impact of congruency between a cartoon character used to endorse a healthy snack with the snack’s characteristics and found that children evaluated the congruent stimuli more positively than incongruent stimuli [70].

### Hypothesis development

Communal wording occurs when words characteristic of and usually associated with stereotypically female behaviours and traits are used. Previous research shows that, on average, women preferred advertisements that were verbal, harmonious, and complex, and men preferred adverts that were comparative (competitive), simple and attribute-oriented suggesting that women and men do differ in their preferences for the styles of advertisements and these differences seem to be explained by the use of specific language or style–more communal for women and more agentic for males [25–27,71].

Given that the message-audience congruence principle suggests that individuals should prefer conditions that are similar to them [40,65,72,73], and homophily theory’s premise that gender is one of the factors that determines one’s sense of similarity to others [74], a leaflet that presents content that is congruent with one’s gender identity should be more effective amongst the respective genders than an incongruent message. if wording is congruent with the endorser’s gender, and this in turn is congruent with respondent’s gender, such leaflets should evoke the most positive responses. Two hypotheses summarise this argument:

**H1:** A leaflet featuring a male endorser and agentic wording will provoke more positive attitude towards advertisement (H1A), and higher behavioural intention among males compared with females (H1B).
**H2:** A leaflet featuring a female endorser and communal wording will provoke more positive attitude towards advertisement (H2A), and higher behavioural intention among females compared with males (H2B).

On the other hand, incongruence between the gendered content of the leaflets (agentic wording endorsed by a female and communal wording endorsed by a male) will not be associated with differences in leaflet evaluations between men and women. Messages which feature wording conceptually incongruent with endorser’s gender will not evoke different evaluations from men and women because the lack of congruence between the different aspects of the message and the respondents.

**H3:** There will be no differences in attitudes towards advert (H3A), and willingness to change behaviour (H3B), between males and females if the endorser is male and the wording is communal.
**H4:** There will be no differences in attitudes towards advert (H4A), and willingness to change behaviour (H4B) between males and females if the endorser is female and the wording is agentic.

## Research methods

This study follows a mixed-methods sequential explanatory design [75]. In such research designs, researchers carry out quantitative and qualitative studies in a consecutive manner, where preceding study informs the research design or selection of variable in subsequent study. Study 2 was carried out as a follow up from Study 1, and the results from Study 2 informed the choice of variables for Study 3 [76]. Structured survey experiments were used in Studies 1and 3, and semi-structured interview employed in Study 2. Study 1 examined effects of gendered content on leaflet effectiveness amongst men and women in the UK. In Study 2, further evidence was sought about how individuals perceive the gendered leaflets to produce richer understanding of responses to gendered content in advertising. Subsequently, informed by results from Study 2, quantitative Study 3 was carried out.

The ethical aspects of the studies have been considered and approved by the following ethics committees: University of Bedfordshire Ethics Committee—BMRI/Ethics/Student/2017-18/001 (Study 1 and Study 2); and University of Bedfordshire Ethics Committee—BMRI/Ethics/Staff/2018-19/006 (Study 3).

Informed participant consent was obtained in all three studies reported in this manuscript. Only adult participants were invited to participate and had to confirm they were 18 years of age or older by ticking a relevant box in the pen-and-paper questionnaire (Study 1 and Study 2), or online questionnaire (Study 3). Please note that the images of individuals featured in S1 and S2 Figs. are used in accordance with the Shutterstock’s Standard Image License which allow the images to be used in digital reproduction. Full terms of the Standard Image License are available here: https://www.shutterstock.com/license. All participants were first provided with the description and nature of the studies, and how data would be used. Then each participant who wished to take part needed to tick a box next to the statement ’Yes, I agree to participate in this study’ on a pen-and-paper questionnaires (Study 1, and Study 2) or online questionnaire (Study 3). After completing the questionnaire, participants were debriefed and given contact details of the first two authors, in case they had questions or comments. No personal data was collected that would enable anyone to identify the respondents. Research data for the three studies is available from LSBU Open Research database in the following links: https://openresearch.lsbu.ac.uk/item/8zz28; https://openresearch.lsbu.ac.uk/item/8zz21; https://openresearch.lsbu.ac.uk/item/8zz27.

### Stimuli development

Leaflets are usually an integral part of real world health-related campaigns, they are easy to distribute (GP offices, community centres, direct leafleting, leaflet handed out by a health professional to people seeking help), and can reach individuals who do not use digital communications [77].

The development of the leaflets followed a process recommended by Geuens and De Pelsmacker [78]. First, we systematically reviewed studies which examined gendered wording to generate a list of gendered words and phrases [79,80]. Simultaneously, a review of actual health promotion campaign materials took place to inform the design of the leaflets. Subsequently, with the help of undergraduate marketing students, two versions of communal wording, and two versions of agentic wording were created. The wording was assessed by a linguist who specialises in gendered wording. A readability analysis was conducted on the text prior to the pre-test. SMOG (simple measure of gobbledygook) test was used and the results showed that the proposed texts would be suitable for readers from the age of 11 and upwards [81].

Next, the four versions of wording were pretested with 33 individuals to select the texts that were perceived to be the most agentic and communal. This was measured on a 5- point semantic differential scale (1-very masculine—5-very feminine). The most feminine text and the most masculine text were then selected. Each leaflet contained similar messages around the positive impact and benefits of walking 30 minutes a day as a physical activity with the overall aim to reduce obesity. Another two questions measured the perceived masculinity/femininity of walking on a 5-point Likert scale. (1-very masculine—5-very feminine). Respondents ranked walking as a gender-neutral activity (M = 3.23, SD = 0.68). Secondly, respondents were asked to rate if doctor was seen as a stereotypically masculine or feminine role. The doctor role was also found to be perceived as gender neutral (M = 3.07, SD = 0.52).

Another pre-test was conducted to select four pictures that were found to be of equal attractiveness. Ten pictures were initially selected from the Shutterstock website. The photos were all similar in terms of characteristics including a dark brown hair colour, endorser stance in terms of arms folded, similar clothing, similar body size, Caucasian ethnicity, with a similar facial expression of smiling and of between the ages of 35–40. A pool of hospital doctors initially chosen (five female and five male) all had the same blue outfits on and included the same characteristics as above. All photos selected were portrait format and on a white background. In the first pre-test attractiveness was measured with a single item measurement on a five-point semantic differential scale (1-very unattractive—5-very attractive). Twenty participants were recruited to test endorsers’ attractiveness. Two images which achieved similar attractiveness evaluations were then selected for the final study.

Next, the name ‘Life training’ was selected. First, a list of brands was generated- the names must have been different from any existing branded health related campaigns and a group of undergraduate marketing students discussed the list of proposed names and voted for one that they perceived the most suitable. The names of the endorsers were selected in the same way: a list of first and last names was generated by undergraduate students. The principles were to avoid very common and uncommon names.

### Final leaflets

Two examples of the leaflets used in the experiments are shown in Appendices 1 and 2. Gendered words for communal wording included in the study were: making the choice, chance, pleasant, gentle, flatterable, understandably, communally, cheerful. Gendered words for the agentic wording included in the study were: decide (making the decision), active, determination, challenging, self-confident, ambitious, individually [26,80]. The amount of gendered wording in each advert was calculated as 3.5% to ensure the wording sounded realistic [26]. The leaflets were designed by a graphic designer and printed at professional printers.

### Study 1

Following a pilot study with 30 participants and minor adjustments (correcting typos and adding clarifications to instructions), the main study took place. Data were collected via a mall intercept survey in the South East of England. Two-hundred-and-forty-seven pen-and-paper questionnaires were collected from November 2017 to March 2018. The survey was distributed in a number of locations and at varying times to ensure that the variability within the population of interest is represented. Hertfordshire was selected as a county to collect data because it resembles an average county with statistics for the percentage of the population identified as either obese or overweight mirroring those for England [82,83].

To participate in the study, the respondents needed to be English speakers who were born, raised, and at the time of the study resided in their homeland. Participants were randomly assigned to one of the four message variations. The participation in the study was voluntary and no financial compensation was offered to the respondents. In order to participate, respondents were informed about the nature and purpose of the study. After initially agreeing, respondents were given a pen-and-paper questionnaire in which they needed to agree to participate in the study by marking ‘yes, I agree to participate in this study’. The same procedure was followed in Study 2 and Study 3 (with Study 3 being an online study, hence, after reading the description of the study, participants had to agree to participate by clicking on the relevant answer).

### Measures

Gender identity is the personal sense of one’s own gender and was measured by one-item categorical question “What is your gender”? (Male, Female) [84].

Attitude towards the advertisement (also referred to as attitude toward ad) is defined as a predisposition to respond in a favourable or unfavourable manner to a particular advertisement during a particular exposure incidence. Attitude towards the advert was measured with six items with a five-point semantic-differential response scale: Irritating/ not irritating, boring/not boring, good/not good, informative/not informative, objective/subjective and appropriate/not appropriate (α = 0.934) [85]. Overall sample M = 4.03 (SD = .92).

Behavioural intention is the willingness to perform a specific behaviour. The participants responded to the statement ‘What is the likelihood you will take up walking 30 minutes a day 5 days a week in the near future?’ There were four 5-point item pairs (Unlikely/ Likely, Improbable/ Probable, Impossible/ Possible, and Uncertain/ Certain) (α = 0.954) [86]. Overall sample M = 3.12 (SD = 1.17).

### Data analysis

The analysis encompassed a twofold data-analytic approach, hence i) descriptive statistics of the sample, and ii) a three-way Multivariate Analysis of Variance (MANOVA) conducted to investigate the interactions between gender (male vs. female), endorser’s gender (male vs. female), wording (agentic vs. communal), and attitude towards the advertising and behavioural intention. The assumptions of the MANOVA analysis were examined in order to determine the suitability of the parametric approach [87]. The data were examined for independency, multivariate normality, univariate and multivariate outliers. Multicollinearity issues were inspected using the Variation Inflation Factors (VIF). The VIF values were ≤ 1.04, which is below the threshold of 10, demonstrating no multicollinearity issues with the data [88]. All statistical analyses were computed with IBM SPSS Statistics Version 26.

### Sample characteristics

In terms of gender, 49.4% (*n* = 122) participants identified themselves as female. The age of the participants ranged from 18 to over 61, and about 25.1% of the participants (*n* = 62) were between the ages of 51 to 60. Majority of participants (80.6%; *n* = 199) identified themselves as English, followed by British (24; *n* = 9.7%) and Scottish (3.6%; *n =* 9), whilst the remaining participants (6.1%; *n =* 15) identified as various other ethnic identities. The majority of the respondents (45.2%, *n* = 87) declared to have A levels/diploma as their highest qualification followed by 22.3% (n = 55) having GSCSE levels, 13.4% (n = 33) had no qualifications, and 18.3% (n = 73) had a university degree. Of our participants 38.5% (*n* = 95) earned between £30,000–£49,999 per year, followed by 30.4% (n = 75) who earned between £10,000-£29,000, and 19.8% (n = 49) earning between £50,000–59,999. The remaining 11.3% (n = 28) earned over £60,000 per year.

No respondents reported to suffer from medical conditions or physical impairments, which could prevent them from walking. In addition, all the respondents had done a minimum of 30 minutes or more of physical activity in the week the study was conducted.

### MANOVA

The MANOVA computations indicated that there was no statistically significant three-way interaction effect of participant’s gender, endorser’s gender, and wording condition on the combined dependent variables (F (2,281) = 2.538, p = 0.081, partial η2 = 0.021.

Further tests revealed main effects of gender, and statistically significant interaction of gender and wording condition (Table 1). Hence, we followed up with Bonferroni pairwise comparisons (Table 2). Table 3 presents descriptive statistics for the outcome measures.

**Table 1:** Main effects and interactions of respondent’s gender, wording condition and endorser’s gender.

Source of variation	Pillai’s Trace	F	Sig.	Partial Eta Squared
***Main effects ***				
Endorser’s gender	0.01	1.71	0.18	0.01
Wording	0.02	2.66	0.07	0.02
Respondent’s gender	0.04	4.60	0.01	0.04
***Interactions***				
Endorser’s gender x Wording	0.01	1.68	0.19	0.01
Endorser’s gender x Respondent’s gender	0.00	0.48	0.62	0.00
Wording x Respondent’s gender	0.08	9.75	0.00	0.08
Endorser’s gender x wording x Respondent’s gender	0.02	2.54	0.08	0.02

Df = 2, error df = 238.

**Table 2:** Pairwise comparisons (estimated marginal means) with Bonferroni adjustments by gender, endorser’s gender, and wording condition.

Dependent Variable	Endorser’s gender	Wording	Respondent’s gender	M	Std. Error	p-value	95% Confidence Interval for Difference	
							Lower Bound	Upper Bound
Behavioural intention	Male	Agentic	Male	3.33	0.29	**0.012**	0.16	1.30
			Female	2.60				
		Communal	Male	3.47	0.30	0.427	-0.35	0.84
			Female	3.23				
	Female	Agentic	Male	3.28	0.29	0.394	-0.32	0.82
			Female	3.03				
		Communal	Male	3.33	0.29	0.059	-0.02	1.14
			Female	2.76				
Attitude towards ad	Male	Agentic	Male	4.32	0.22	**0.000**	0.55	1.41
			Female	3.34				
		Communal	Male	3.86	0.23	0.056	-0.89	0.01
			Female	4.30				
	Female	Agentic	Male	4.13	0.22	0.209	-0.15	0.70
			Female	3.85				
		Communal	Male	4.17	0.22	0.444	-0.61	0.27
			Female	4.35				

**Table 3:** Descriptive means.

Sources of variation			Behavioural intention		Attitude towards ad	
Endorser’s gender	Wording	Respondent’s gender	Mean	SD	Mean	SD
Male	Agentic	Male (N = 31)	3.33	1.28	4.32	1.00
		Female	2.60	1.13	3.34	0.93
		(N = 33)				
	Communal	Male (N = 30)	3.47	1.30	3.86	0.82
		Female	3.23	1.06	4.30	0.94
		(N = 28)				
Female	Agentic	Male	3.28	0.98	4.13	0.88
		(N = 33)				
		Female	3.03	1.14	3.85	0.92
		(N = 31)				
	Communal	Male	3.33	1.23	4.17	0.76
		(N = 31)				
		Female	2.76	1.09	4.35	0.69
		(N = 30)				

When exposed to agentic wording presented by a male endorser, men declared more positive attitude towards ad (M = 4.32) than women (M = 3.34), SE = 0.22, p = .000; and declared higher behavioral intention (BI) (M = 3.33) than women (M = 2.60), SE = 0.29, p = 012, hence hypotheses H1A and H1B are supported.

When exposed to communal wording presented by a female endorser, women declared more positive attitude towards ad (M = 4.35) than men (M = 4.17; SE = 0.22) but the difference was not statistically significant (p = 0.444). Women declared lower BI (M = 2.76) than men (M = 3.33) but the difference was not statistically significant. Hypotheses H2A and H2B are not supported.

When presented with a communal wording endorsed by a male, women declared more positive attitude towards ad (M = 4.30) than men (M = 3.86, SE = 0.23) but this difference was not statistically significant (p = .056). and on the BI measure, men declared higher BI (M = 3.47) than women (M = 3.23, SE = 0.30) but it was not statistically significant (p.0.427). H3 is therefore supported.

When presented with agentic wording endorsed by a female, men declared more positive attitude towards ad (M = 4.13) than women (M = 3.85) but the difference was not statistically significant (p = 0.209), and men declared higher BI (M = 3.28) than women (M = 3.03) but it was not statistically significant (p = 0.0394). H4 is therefore supported.

**Fig 1:**
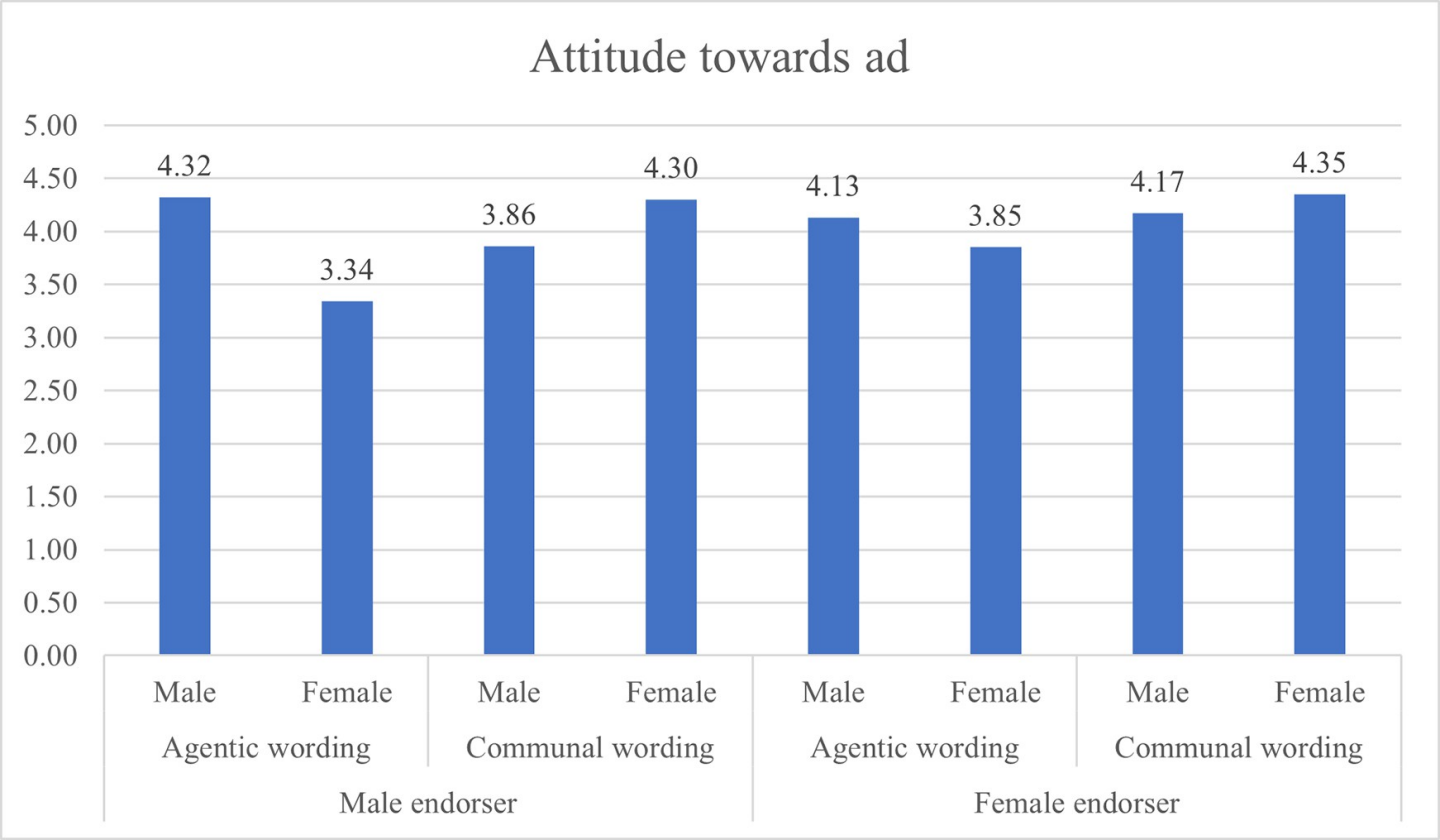
Attitude towards ad ratings based on gendered content and respondents’ gender.

**Fig 2:**
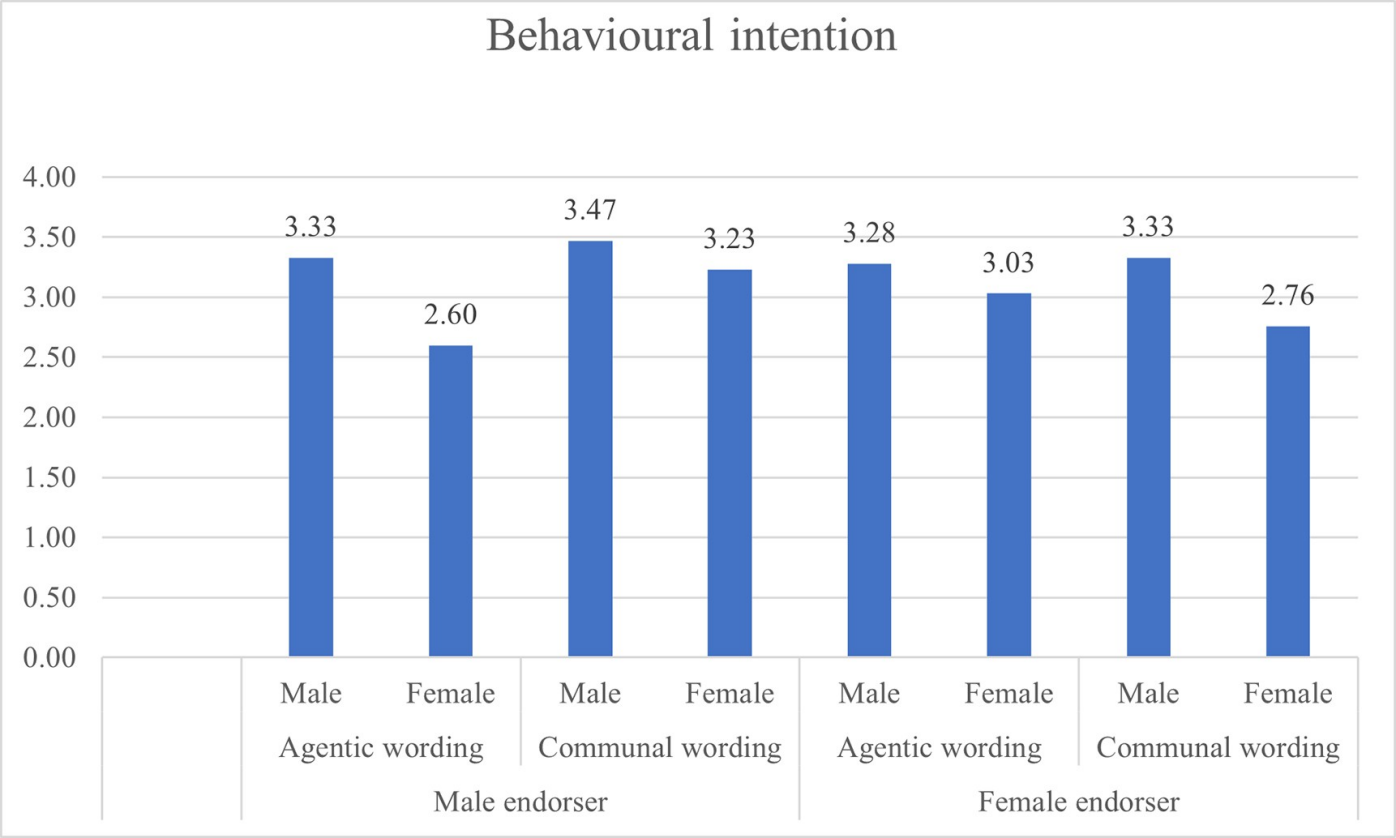
Behavioural intention ratings based on gendered content and respondents’ gender.

Figs 1 and 2 present the attitude towards ad and behavioral intention evaluations grouped by gender.

### Study 2: Semi-structured interviews

Twenty individuals (ten males and ten females) were randomly selected from those who during Study 1 expressed an interest by volunteering their contact details to the researcher in taking part in the semi-structured interviews. The interviews took place between March 2018 and July 2018.

### Interview guide and leaflets

A leaflet with agentic wording and featuring a male endorser and a leaflet featuring communal wording and a female endorser were used in the interview to further explore respondents’ opinions, attitudes and behavioural intentions stimulated by the chosen leaflets. The two leaflets were chosen as these were guided by the results from the first study: two leaflets that had the highest attitude towards ad scores were used, also because during pilot study when all four leaflets were used, respondents found it too laborious. Respondents instead were asked to imagine how they would perceive a leaflet if it had a male/female endorser combined with the wording option. The interview began by asking respondents general questions about their exercise habits, and then were presented with the two leaflets to discuss their perceptions, opinions and attitudes towards the presented leaflets.

### Data analysis

We applied the principles of directed qualitative content analysis as described by [89] combined with deductive coding [90] in order to identify patterns in responses to leaflets from men and women. The coding’s aim was to compare responses from men and women, so we initially divided the transcripts into two groups, each containing transcripts from one gender. The next step was to code the sentiment and quantify the positive and negative responses by gender. Then we identified reasons given for the positive and negative evaluations. Data from the semi-structured interviews was analysed following the procedure suggested by [91]. The data available were typed up in a Word document. The first researcher analysed the transcripts and made annotations, then a second researcher analysed the transcripts in a separate document following the same procedure as the first researcher. The coding then was analysed, discussed and refined by a third researcher to resolve any differences in coding. Three main patterns emerged from the interview data: 1) the majority of male respondents preferred agentic wording presented by male endorser (wording–endorser–audience congruence for male participants); 2) a majority of females did not express a very strong preference for one or the other (message-respondent neutrality for female participants); 3) a minority of males (females) expressed a preference for communal (agentic) wording (message-respondent incongruence). Below, we briefly summarise the patterns and illustrate with selected quotes.

1. Wording–endorser–audience congruence for male participants. The majority of interviewed males expressed a preference towards the leaflet featuring a male endorser and agentic wording and they pointed at the wording as the aspect of the leaflet that attracted them to it. One of the male participants said *“It suits my sort of character more so*, *really*.*”* Another respondent said: *“I know it’s this one*. *It’s just the wording’s a little bit different and I think that could have an impact without you really noticing that much*. *For example*, *making the decision*, *making the choice*.*”* Yet another male participant said *“I think it’s the male thing because for me that’s kind of from a gender point of view*, *I can relate more*, *and so I can think*, *okay*, *that’s something I would take on board*.*”* He continued after pointing at leaflet 2 (female endorser and communal wording) *“if I saw this*, *I would think it would relate to women*.*” […]I wouldn’t have taken that as directly related to me*, *even though it doesn’t mention gender on here*.*”* Another male participant expressed a preference for the first leaflet because of the more direct expressions *“I’m happy for more direct type of information*.*”* Another participant’s perspective on the same leaflet was expressed by emphasising the words and phrases that stood out to him (participant read aloud the phrases in the exact order as presented here): *“This is far more factual*. *Far more blunt*…*"*
  Another reason why most males liked leaflet 1 (male endorser/agentic) was the male endorser: *“I think both the leaflets are important*, *because one speaks to the women and the other one speaks to the men…it’s somebody from my own gender who is recommending it*. *But I personally feel you need two leaflets*, *one for women*, *and one for men*.*”* Another male participant said *“I could actually see blokes with a bloke on there would prefer*, *and women with a woman on there*. *It’s like women tend to see women doctors*.*[*..*] I can see individuals picking their sex on there*.*”* In the two aforementioned examples, the male respondents express their views that men and women are different, and both need different approaches to communication. This suggests they think messages should be similar to the audience they are targeting, and that men and women require different messages, suggesting that congruence is important.
  Another male participant said *“This one seems more like a nurse*. *This one looks like a practicing GP*, *he looks more striking*. *That’s not coming across a sexist*. *But it just looks more affirming*.*”* A second male participant said *“Okay*, *it’s not even in like really*… *like I’m not trying to be sexist or anything like that*. *I feel like the image*… *I don’t know why*, *again I think this image is stronger*, *the pose*.*”*
2. Message-respondent neutrality for female participants. The majority of female participants in this study did not express a strong preference for either leaflet. *“I don’t know*. *Either*, *I would say*. *It wouldn’t bother me if it was coming from a male or a female*.*” Another participant said”I don’t actually think I have a preference*, *to be honest*. *It doesn’t particularly bother me whether there’s a male or a female giving the information*.*”* However, the few females that did prefer leaflet 2 gave the following insights *“it’s more chatty*. *It’s more of a female way of talking if that makes sense*.*”* And continued: *“It’s just the words because it’s more like the lady doctor is talking so it’s more of a feminine way of speaking*. *[…] It’s just a gentler voice whereas with the man*, *it feels just a bit more pushy*.*”* As evidenced in the previous quote, some women also assigned different characteristics to men (pushy) and women (feminine way of speaking). Another woman preferred leaflet number 2 because *“it’s less clinical*, *it feels more personal*. *and it’s easier to sort of read it because it’s like someone actually cares about it…*… *Because it’s less like being told what to do and this makes it feel like it’s easier and it’s more gentle and it’s more like someone’s having a chat with you that actually cares rather than a doctor saying you have to do this or you should do that*.*”*
3. Message-respondent incongruence. In terms of the wording for leaflet 2 (female endorser/ communal wording) some perspectives were given from the small number of male participants who preferred leaflet two suggesting that incongruity between the wording and the endorser’s gender was not perceived as less effective to them: *“I think*, *actually*, *in the one with Julie*, *I think actually the actual wording of the text*, *though*, *I think does make it*… *It softens it slightly”* and further elaborated that the mention of mental and emotional health was the reason the leaflet struck *“a chord”* with him, because of his *“own issues with my*, *let’s say depression*.*”* However, some words were seen as ‘too much’ even for this male who preferred communal wording: *“Apart from*, *for me*, *the "flatterable physique*.*" That seems very feminine*.*”* Another male participant said *“I mean*, *if I was to choose from one or two*, *I think number two would be*… *it’s more*… *it’s easy to read*.*”*

Based on the insights from the interviews which show participants used words such as ‘feminine’, ‘gentle’, ‘more personal’, ‘caring’ (to describe the communal wording and female endorser), ‘masculine’, ‘pushy’, ‘affirming’ (to describe the agentic wording and male endorser) to describe their perceptions of the leaflets, this suggests that respondents assigned different characteristics to the endorsers. The words and phrases that respondents used to describe leaflets suggest they perceive men and women to have different roles. Following from this, we therefore included the respondent’s gender role identity as a construct that may impact the evaluations of gendered content. The gender role identity measure assesses to what extent one identifies with descriptions of characteristics that are stereotypically associated with men and with women [92].

Moreover, we included another measure of effectiveness, namely ad credibility as many interview participants specifically mentioned that some wording sounded more credible to them.

### Study 3: UK general population

Drawing on findings from study 2, in study 3 we examine the role of gender role identity on the effectiveness of the gendered leaflets. Study 3 approaches gender differences from a psychological perspective and proposes that the individual’s gender role identity (masculinity and femininity) is related to how they evaluate gendered content of leaflets. Whilst study 1 approached gender from a perspective of a general gender identity, in study 3 we measure how one identifies with characteristics that are commonly viewed as related to females and males [92,93]. Gender role identity is defined as the all-important characteristics and personality tendencies that stereotypically are seen as differentiating females from males including attributes related with feminine traits (e.g. sensitive, affectionate, gentle, nurturing, emotional) and masculine traits (e.g. ambitious, forceful, instrumental, competitive) that are evident within each person [92]. A person usually displays all of these traits but to different degrees. Some individuals may be more masculine–that is, display more characteristics associated stereotypically with males (masculine traits), and others may display traits stereotypically associated with women (feminine traits). In yet other individuals, these traits may exist in a more balanced way.

As in study 1, we draw on the homophily theory and congruency concept to suggest that when there is congruence between the gendered content of the leaflet and the characteristics of the respondent (gender and gender role identity), this will lead to more positive evaluations of the leaflets. Hence the following hypotheses are proposed:

**H5.** There will be an interaction between endorser’s gender, wording condition, gender role identity and participant’s gender on attitude towards ad (H5a), behavioural intention (H5b) and advert credibility (H5c).
**H6:** A leaflet featuring a male endorser and agentic wording will provoke more positive attitude towards advertisement (H6A), more positive behavioural intention (H6B), and more positive advertising credibility (H6C) among masculine males compared with masculine females.
**H7:** A leaflet featuring a female endorser and communal wording will provoke more positive attitude towards advertisement (H7A), more positive behavioural intention (H6B), and more positive advert credibility (H6C) among feminine females compared with feminine males.
**H8:** There will be no differences in attitude towards advert (H8A), behavioural intention (H8B), and advert credibility (H8C) between feminine /masculine males and females if the endorser is male and the wording is communal.
**H9:** There will be no differences in attitude towards advert (H9A), behavioural intention (H9B), and advert credibility (H9C) between feminine/masculine males and females if the endorser is female and the wording is agentic.

### Study 3: Method

#### Participants and procedures

For Study 3, respondents were recruited in the UK using the online survey platform Qualtrics.com. The eligibility of respondents was aligned with Study 1, hence respondents needed to be English speakers who were born, raised, and resided in the UK. The same four message variations were used in the survey. The survey distribution was randomized to ensure an even sample of men and women across the different manipulations. In addition to the authors of the study, a research assistant (RA) was employed to collect data. The RA, due to her previous employment history, had large network of personal contacts who were past customers of a temporary employment agency. The research team posted the invitation to the study on social media platforms and used personal contacts to collect surveys via snowballing. In addition, students were asked to distribute the link to the survey amongst their friends, and family members. This procedure was repeated several times over 6 months in order to reach a sufficient sample size to meet the requirements of the intended statistical tests. A total of 726 surveys were collected between January and June 2019. Incomplete surveys were excluded using the listwise deletion approach, then responses which failed the attention check (measured with the following sentence: “I *fly to the moon every day”)* were excluded resulting in 599 fully completed surveys with close distribution between the four stimuli and genders of respondents.

#### Measures

The survey was identical to the one used in Study 1 with the addition of variables identified during Study 2, namely advertising credibility and dominant gender role identity (DGRI).

Advertising credibility was measured with three items anchored with a seven-point Likert scale ranging from 1 = “Strongly disagree” to 7 = “Strongly agree” [41]. Average scores were obtained by summing the answers for each participant, and dividing it by the number of items (3) with higher scores being indicative of higher credibility towards the advertising (α = 0.917). Overall sample M = 5.22 (SD = 1.56).

Attitude towards ad was measured with the same items as in study 1 (α = .914, M = 5.29, SD = 1.410; and so was behavioral intention (α = .954, M = 2.95, SD = 1.15). Dominant gender role identity was captured using the Gender Trait Index scale (GTI) [71]. The scale considers femininity and masculinity as two distinct dimensions that co-exist in varying levels within an individual. It measures gender role identity at the individual level with 16 items describing feminine and masculine characteristics.

Femininity is measured using eight items, which captures self-reported perceptions such as affection, tenderness, sensitivity to others’ needs, sympathy, warmth, eagerness to soothe hurt feelings, gentleness, and compassion (α = 0.95). Masculinity is measured using eight items reflecting self-reported perceptions in terms of having leadership abilities, assertiveness, willingness to take a stand, ambition, competitiveness, strong personality, forcefulness, and act like a leader (α = 0.88). In this study, both scales were rated using a 5-point Likert scale ranging from 1 = “Never” to 5 = “Always”. Total scores for each of the GTI dimensions were obtained by summing the answers, which could range from 8 to 40, with higher scores indicating higher levels of self-reported femininity and masculinity. The final scores on the GTI indicated either higher levels of masculinity, higher levels of femininity or neutral if the levels of femininity and masculinity were equal. If the total scores for femininity were found higher than masculinity, then femininity was the DGRI (coded as 1). If the total score for masculinity were found higher than femininity, then masculinity was the DGRI (coded as 2). DGRI was labelled as neutral if masculinity and femininity scores were equal (coded as 0)

Finally, the consumer’s attitudes towards the advertising and the consumers’ willingness to change behaviour yielded consistent internal reliability (α = 0.914, and α = 0.954 respectively).

#### Data analysis

Similar to Study 1, the assumptions of the MANOVA and relevant preliminary analysis were examined to determine the suitability of the parametric approach. Following the examination of the data, the assumptions of normality and homogeneity of variance have been violated, and hence Pillai’s trace has been employed as a test statistic more robust to such violations [94]. Following this, we also reported Pillai’s trace in Study 1 to maintain consistency of test statistics across the two studies.

### Results

#### Sample characteristics

The structure of the sample is as follows: females (50.9%; *n* = 305), the age ranged from 18 to 75 (Mean_age_ = 38.83, *SD* = 11.87), white ethnicity (89.3%; *n* = 535), and working full-time (71.4%, *n* = 425). Similar to Study 1, no respondents reported to have any medical conditions or physical impairments, which could prevent them from walking. About 84.6% (*n* = 507) respondents had undertaken a minimum of 30 minutes or more of physical activity in the week the study was conducted. There were no statistical differences in the patterns of responses of those who declared to partake or not in any physical activity.

#### MANOVA

The output of the four-way MANOVA showed that there was a significant interaction effect between the participant’s gender, endorser’s gender, communal/agentic condition of the advertising, and DGRI on the combined dependent variables, F(3, 581) = 24.052, p = .000; Pillai’s Trace = .110, *partial η*^*2 =*^ .*110*. In addition, the remaining main and interaction effects are presented in Table 4.

**Table 4:** Four-way multivariate analysis of variance (MANOVA) for the three sets of outcome measures: Behavioral intention, ad credibility, and attitude towards ad.

*Source of variation*	Pillai’s Trace	F*	p-value	Partial *η*^*2*^
***Main effects***				
Endorser’s gender	0.02	5.13	0.002	0.02
Wording	0.01	3.01	0.029	0.01
Respondent’s DGRI	0.06	12.29	0.000	0.06
Respondent’s gender	0.09	20.12	0.000	0.09
***Interactions***				
Endorser’s gender x Wording	0.02	5.41	0.001	0.02
Endorser’s gender x Respondent’s DGRI	0.05	10.12	0.000	0.05
Endorser’s gender x Respondent’s gender	0.03	6.92	0.000	0.03
Wording x Respondent’s DGRI	0.09	20.58	0.000	0.09
Wording condition x Respondent’s Gender	0.25	64.65	0.000	0.25
Respondent’s DGRI x Respondent’s gender	0.00	.72	0.539	0.00
Endorser’s gender x Wording x Respondent’s DGRI	0.02	4.01	0.008	0.02
Endorser’s gender x Wording x Respondent’s gender	0.02	4.24	0.006	0.02
Endorser’s gender x Respondent’s DGRI x Respondent’s gender	0.04	8.89	0.000	0.04
Wording x Respondent’s DGRI x Respondent’s gender	0.13	30.75	0.000	0.13
Endorser’s gender x Wording x Respondent’s DGRI x Respondent’s gender	0.11	24.05	0.000	0.11

*Df = 3, DF error = 581.

For the next stage of the data analysis, a four-way MANOVA was carried out to test Study 3 hypothesis. The computations supported a significant interaction between the participant gender, DGRI, endorser’s gender, and wording on advertising credibility (**H5c**) (*F* (1, 599) = 20.10, *p =* 0.000, *partial η*^*2*^ = 0.33 (**H5a**) and on attitude towards advertisement (*F* (1, 599) = 9.60, *p =* 0.002, *partial η*^*2*^ = 0.03); and on behavioural intention (**H5b**) (*F* (1, 599) = 29.71, *p =* 0.000, *partial η*^*2*^ = 0.04).

The *post-hoc* pairwise comparisons were computed with the Bonferroni test. The detailed pairwise comparisons are presented in Table 5. Pairwise comparisons revealed that when exposed to agentic wording presented by a male endorser, males with masculine DGRI rated it higher (M = 6.37, SE = 0.27) than females with masculine DGRI (M = 4.46, SE = 0.27), p = .000 on advertising credibility (H6C), and on attitude towards ad (Males: M = 6.13, SE = 0.26; females: M = 4.69, SE = 0.26), p = 0.000 (H6A), and behavioural intention (males: M = 3.03, SE = 0.25, females: M = 2.86, SE = 0.25) but this difference was not statistically significant (p = 0.503). Hence H6 is partially supported as congruence led to higher evaluations of the leaflet only for two measure of leaflet effectiveness.

**Table 5:** Pairwise comparisons by respondent’s gender, dominant gender role identity (DGRI), endorser’s gender, and wording condition.

Dependent Variable	Endorser’s gender	Wording condition	Respondent’s DGRI	Respondent’s gender	Mean	Std. Error	p-value	95% Confidence Interval for Difference	
								Lower Bound	Upper Bound	
Attitude towards ad	Female	Communal	Femininity	Male	5.36	0.25	**0.001**	-1.36	-0.35
				Female	6.22				
			Masculinity	Male	3.65	0.25	**0.000**	-2.95	-1.95
				Female	6.10				
		Agentic	Femininity	Male	6.04	0.26	**0.029**	0.05	1.09
				Female	5.46				
			Masculinity	Male	5.80	0.25	**0.001**	0.34	1.34
				Female	4.96				
	Male	Communal	Femininity	Male	5.34	0.26	**0.001**	-1.40	-0.35
				Female	6.21				
			Masculinity	Male	3.38	0.26	**0.000**	-3.25	-2.21
				Female	6.11				
		Agentic	Femininity	Male	4.42	0.25	**0.001**	-1.35	-0.33
				Female	5.27				
			Masculinity	Male	6.13	0.26	**0.000**	0.92	1.95
				Female	4.69				
Advert credibility	Female	Communal	Femininity	Male	5.12	0.27	**0.000**	-1.70	-0.64
				Female	6.29				
			Masculinity	Male	3.36	0.26	**0.000**	-2.87	-1.82
				Female	5.71				
		Agentic	Femininity	Male	5.74	0.27	0.34	-0.28	0.81
				Female	5.47				
			Masculinity	Male	6.05	0.26	**0.000**	0.54	1.58
				Female	4.98				
	Male	Communal	Femininity	Male	5.24	0.28	**0.001**	-1.46	-0.35
				Female	6.14				
			Masculinity	Male	2.83	0.27	**0.000**	-4.21	-3.11
				Female	6.49				
		Agentic	Femininity	Male	4.44	0.27	**0.004**	-1.31	-0.24
				Female	5.22				
			Masculinity	Male	6.37	0.27	**0.000**	1.37	2.45
				Female	4.46				
Behavioural intention *	Female	Communal	Femininity	Male	3.37	0.25	0.614	-0.36	0.61
				Female	3.24				
			Masculinity	Male	2.33	0.24	**0.004**	-1.19	-0.22
				Female	3.05				
		Agentic	Femininity	Male	1.81	0.25	**0.000**	-1.89	-0.88
				Female	3.20				
			Masculinity	Male	3.36	0.24	**0.003**	0.25	1.22
				Female	2.62				
	Male	Communal	Femininity	Male	3.62	0.26	0.942	-0.49	0.52
				Female	3.60				
			Masculinity	Male	2.31	0.25	0.702	-0.60	0.40
				Female	2.41				
		Agentic	Femininity	Male	3.92	0.25	**0.000**	0.72	1.71
				Female	2.69				
			Masculinity	Male	3.03	0.25	0.503	-0.33	0.67
				Female	2.86				

*Behavioural intention was measured on a 1–5 scale.

When exposed to communal wording presented by a female endorser, females with feminine DGRI rated the leaflet higher on attitude towards advertising (M = 6.22, SE = 0.25) than feminine males (M = 5.36), p = 0.001 (H7A); advert credibility (females M = 6.29); males M = 5.12), p = 0.000 (H7B) but not on behavioural intention (females M = 3.24, males M = 3.37), p = 0.614 (H7C). H7 is partially supported.

When presented with communal wording and male endorser, contrary to our hypotheses, statistically significant differences were found for attitude towards ad (H8A), and advert credibility (H8C), but no statistically significant differences for behavioural intention (H8B).

**Fig 3:**
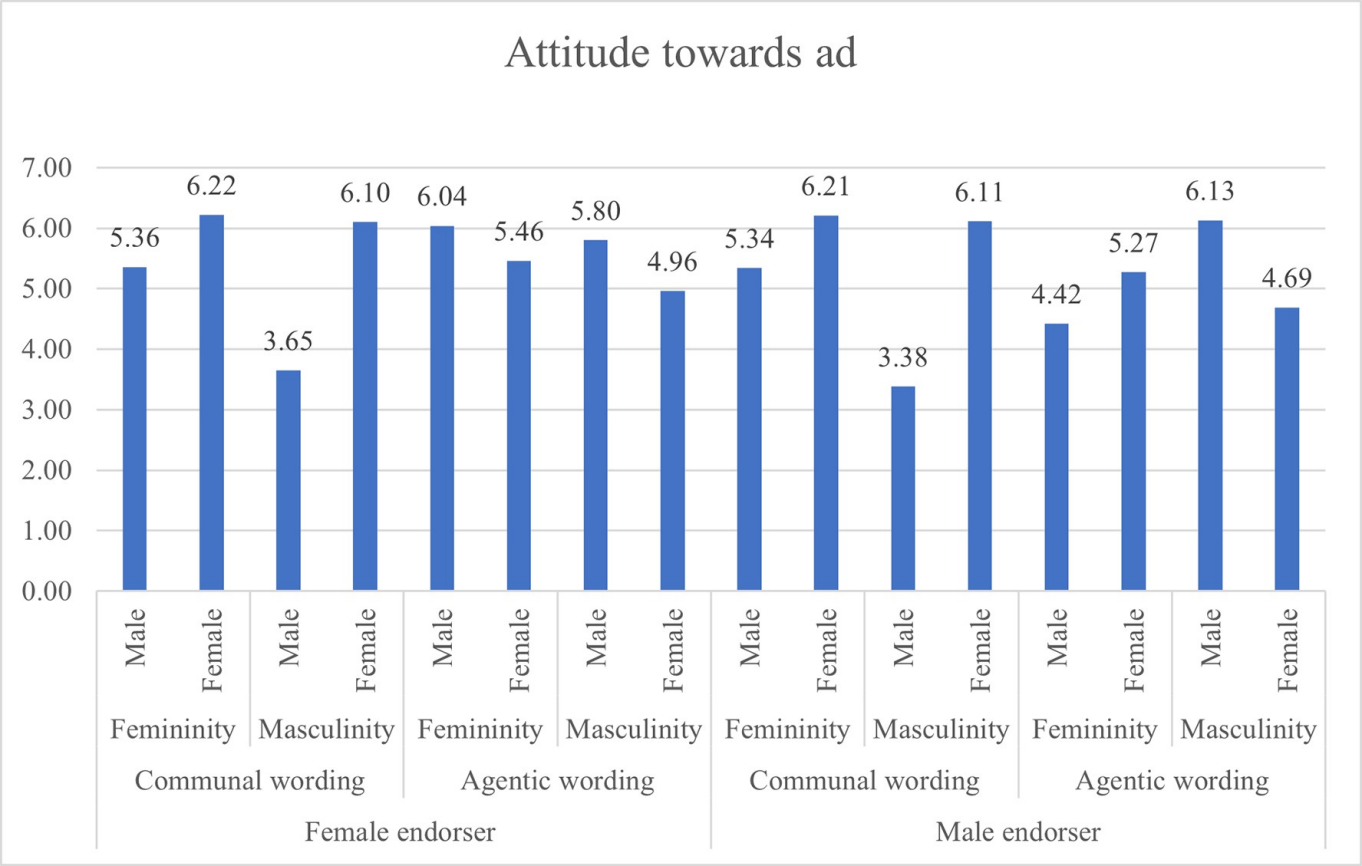
Attitude towards ad ratings based on gendered content and respondents’ DGRI and respondents’ gender.

**Fig 4:**
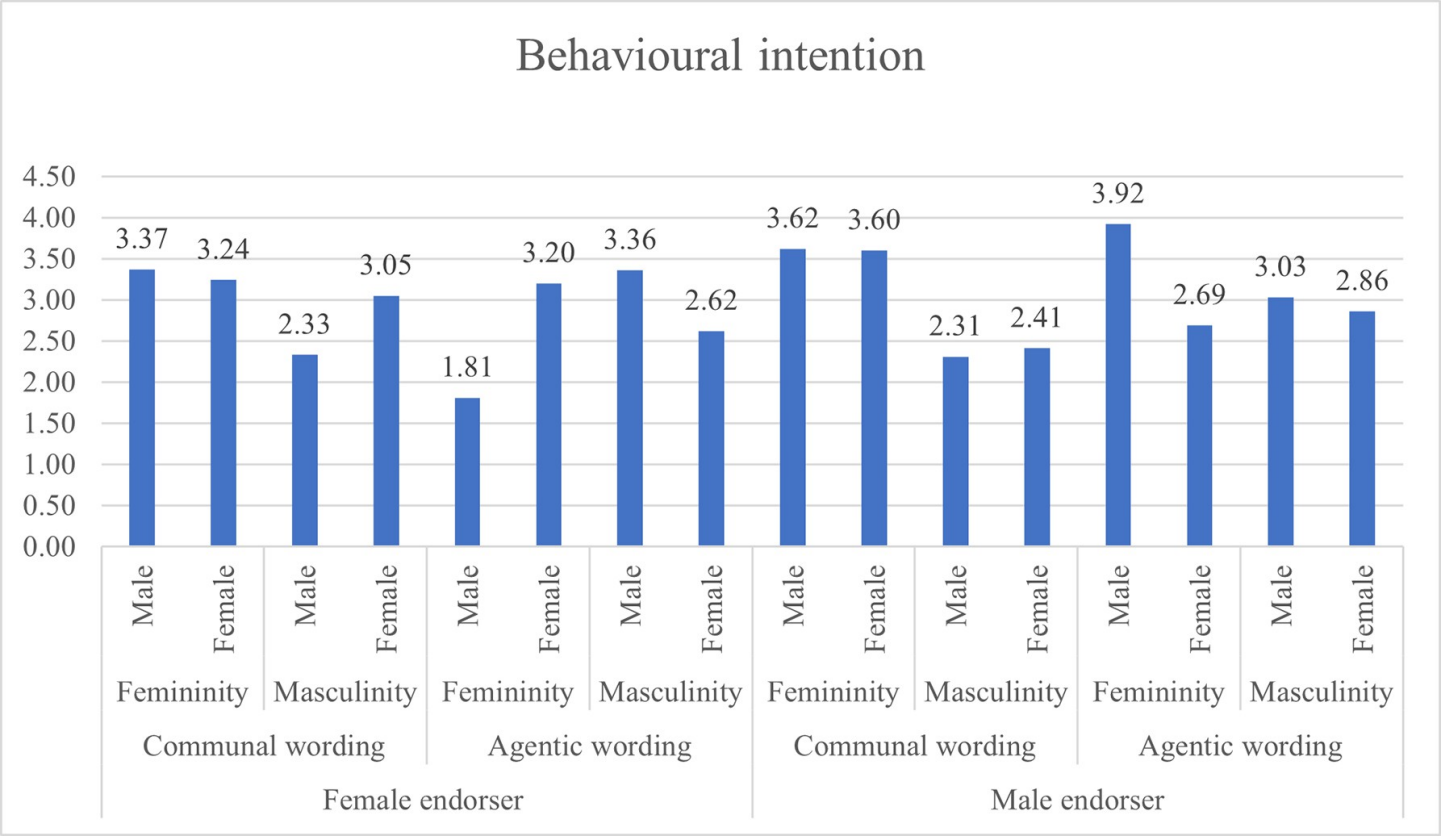
Behavioural intention ratings based on gendered content and respondents’ DGRI and respondents’ gender.

**Fig 5:**
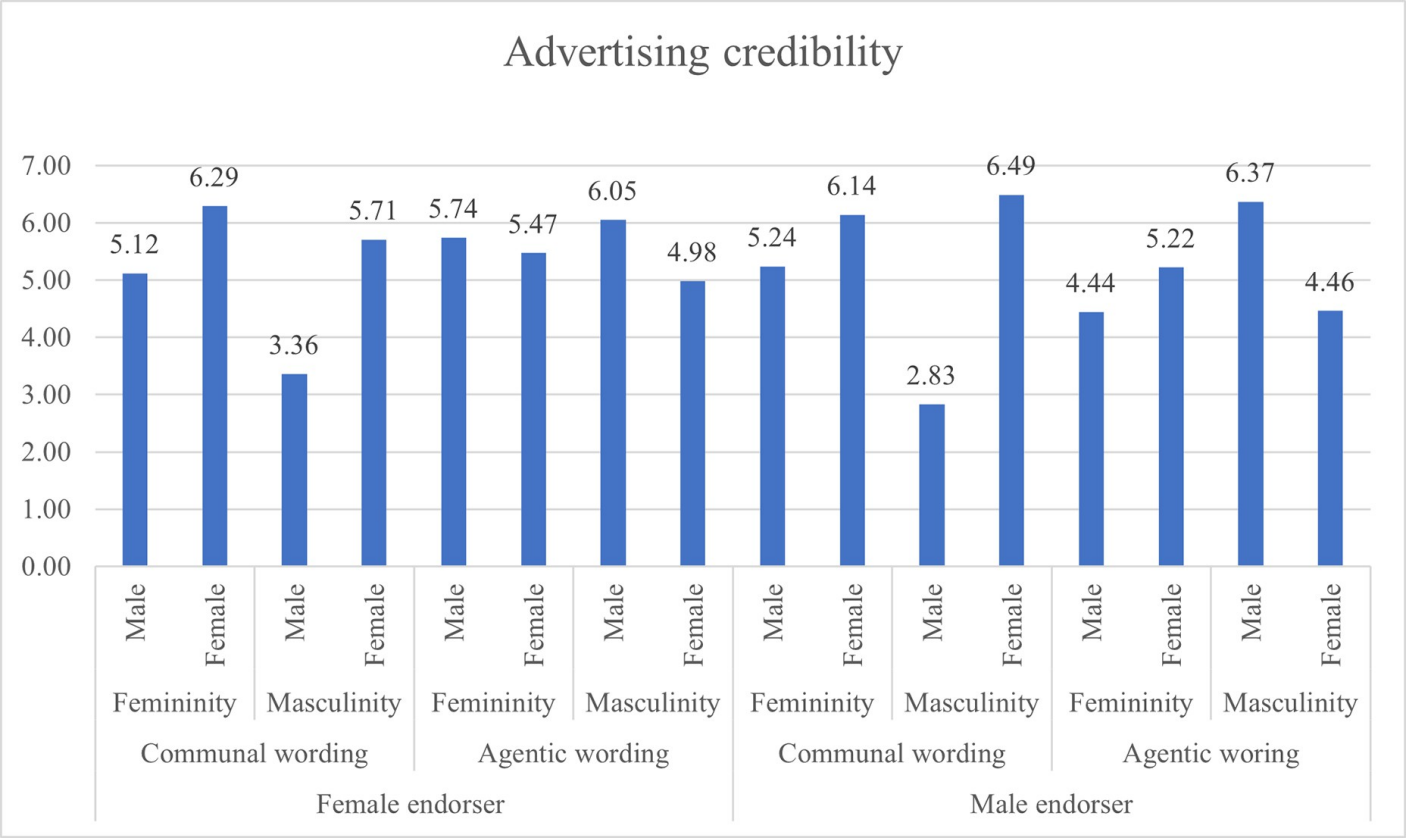
Advertising credibility ratings based on gendered content and respondents’ DGRI and respondents’ gender.

When presented with agentic wording presented by a female endorser, masculine males rated the leaflet higher than masculine females for advert credibility (p = .000), feminine males rated it higher than feminine females (p = .029), and masculine males rated it higher than masculine females (p = .001) for attitude towards ad (H9A), and for willingness to change behaviour masculine males rated it higher than masculine females (p = 003), and feminine females higher than feminine males (p = .000). Other paired comparisons were statistically not significant. Figs 3, 4 and 5 present the attitude towards ad, behavioural intention and advertising credibility ratings based on gendered content and respondents’ DGRI and gender.

The observed means regarding the participant gender, endorser’s gender, wording condition of the advertising on attitude towards ad, ad credibility, and behavioural intention are presented in Table 6.

**Table 6:** Observed means for attitude towards ad, ad credibility, and behavioural intention.

Endorser’s gender	Wording	Respondent’s DGRI	Respondent’s gender	Ad credibility		Attitude towards ad		Behavioural intention	
				M	SD	M	SD	M	SD
Female	Communal	Femininity	Male (N = 29)	5.12	1.10	5.36	0.84	3.37	1.44
			Female (N = 45)	6.29	0.99	6.22	1.11	3.24	1.20
		Masculinity	Male (N = 48)	3.36	1.19	3.65	0.77	2.33	0.69
			Female (N = 29)	5.71	0.70	6.10	0.71	3.05	0.60
	Agentic	Femininity	Male (N = 26)	5.74	1.02	6.04	0.64	1.81	1.18
			Female (N = 46)	5.47	1.48	5.46	1.36	3.20	0.86
		Masculinity	Male (N = 44)	6.05	0.52	5.80	0.54	3.36	1.00
			Female (N = 31)	4.98	1.44	4.96	1.17	2.62	1.05
Male	Communal	Femininity	Male (N = 25)	5.24	1.12	5.34	1.40	3.62	0.84
			Female (N = 47)	6.14	1.00	6.21	1.08	3.60	1.00
		Masculinity	Male (N = 49)	2.82	1.36	3.38	1.12	2.31	0.90
			Female (N = 25)	6.49	0.44	6.11	0.66	2.41	0.79
	Agentic	Femininity	Male (N = 25)	4.44	1.00	4.42	0.48	3.92	0.95
			Female (N = 56)	5.22	1.30	5.27	1.66	2.69	1.25
		Masculinity	Male (N = 48)	6.37	0.74	6.13	0.78	3.03	1.27
			Female (N = 26)	4.46	1.71	4.69	1.43	2.86	1.24

## General discussion

Drawing on homophily theory and the message-audience congruence concept, this study examined the effectiveness of gendered wording and endorser’s gender in a health promotion leaflet by conducting three studies in the UK. We extend previous gendered communication research by examining the use of gendered content in a new communication context, i.e. health promotion, and in a sample not previously studied in gendered wording context. Therefore, we provide additional evidence about the effectiveness of such message strategies at a time when gender roles have been changing in British society. Although the effectiveness of gendered wording has been examined in many countries, the UK has not featured in this research context so far. As gender and gender identity issues continue to be of importance in British society, and continue to receive policy and media attention, we argue it is important to examine the effects of gendered wording in this selected communication context.

In Study 1, the statistically significant results showed males reported higher scores when the wording was agentic and the endorser was a male compared with females, for both behavioural intentions and attitude to advert. Interestingly, however, when looking more closely at the scores we found that men reported higher behavioural intention than women for all leaflets, but for attitude towards ad, the evaluations did not follow this pattern. Women evaluated communal wording more positively than men, and men evaluated agentic wording more positively than women. These findings were largely confirmed in the qualitative study, in which the majority of male respondents preferred a leaflet featuring a male endorser and agentic wording, and the majority of females reported neutral responses to the presented leaflets.

Study 3 showed that men with masculine DGRI evaluated communal wording negatively but agentic wording positively (regardless of the gender of the endorser). Masculine men were the only group who evaluated communal wording negatively. Other respondents (feminine males and feminine/masculine women), whilst they did differ in their evaluations of the four leaflets, evaluated all of the leaflets positively—regardless of the gendered content. Study 3 provided more clarity to men’s responses and suggests that, in men, it is the level of masculinity that is related to their responses to gendered wording. This again follows the pattern from Study 1 and Study 2 and confirms that the wording of a leaflet needs to be considered more carefully when targeting masculine males, providing support for the existing assumptions of homophily and the message-audience congruence concept for a male audience.

The findings of this study suggest that gender role expectations may be changing [74], and women are now supported to take on roles and behaviours that were in the past only acceptable for men. Women are now socialised to pursue a variety of roles and encouraged to break gender stereotypes, so women may respond positively to both masculine and feminine wording, regardless of their dominant gender role identity. This may explain why agentic wording presented by a male in Study 1 achieved positive behavioural intentions amongst both men and women, and the majority of the women in Study 2 did not show any specific preference for one wording over the other.

Research shows that women have been moving into traditionally male, agentic occupations but this shift has not been as visible for men [95,96]. The balance of communal and agentic traits within females may be changing so they can adapt to these job roles with more women in recent years working in careers requiring authority and power [27]. In other words, females may have adopted certain agentic traits due to changes in their occupational positions [97]. Therefore, this study challenges the common assumptions of the message-audience congruence concept and homophily theory for females in relation to gendered wording and the endorser’s gender. As gender roles and societal gender role expectations are changing, women are now encouraged to take on stereotypically male roles and professions, and/or to display behaviours stereotypically reserved for men in the past [98]. Therefore conceptual congruence may not be an accurate construct that explains the persuasion effects of gender-based message content, and so factors such as perceived congruence should also be examined in future studies [99].

Our findings stand in opposition to some past research which claimed that women are discouraged by agentic wording, albeit such findings relate to the context of job ads and in non-UK samples. For example, Oldford and Fiset [12] found women were more likely to apply for finance jobs when the advert featured communal wording and discouraged when the job advert contained agentic wording.

Moreover, our findings stand in opposition to studies which demonstrated that men did not differentiate between masculine and feminine wording [27]. However, findings from studies which were carried out outside of the UK and focused on job adverts may not apply to an activity that is relatively neutral (walking) and a British sample [100]. As culture and language may affect behaviour, our UK-based findings need to be validated in further studies with British respondents.

Theoretically, we identified that gender-based message-respondent congruence is not a necessary aspect of communications to be effective, except for one group: masculine males. Our study identified dominant gender role identity as a construct that explained respondents’ preferences for presented stimuli. Specifically, males who display masculine gender role identity differ in evaluations of communal wording from all other groups. Further research should focus on exploring the processes underlying these responses and examining why conceptual congruence matters for masculine males but not for other groups. It may be related to cultural congruency [69,101], perhaps explaining why our findings do not follow the patterns demonstrated in gendered wording studies conducted outside of the UK.

This study has important practical implications for advertisers and social marketers who wish to use gendered content in their communications strategies. The findings may serve not-for-profit organisations (such as local government public health departments or social marketing advertisers) as well as commercial organisations (such as gyms) to target men and women more effectively by employing gendered wording. Public health and commercial campaigns could use male endorsers with agentic wording for masculine-focused physical activity campaigns.

However, it needs to be noted that if employing such gendered language, one needs to consider the efforts to introduce ‘gender-fair’ language [102] and how such use helps to maintain social expectations around gender.

### Limitations and future research

The results of the three studies should be viewed with their methodological boundaries and limitations. Although survey experiments and semi-structured interviews are appropriate methods to study message effectiveness, such methods can raise concerns about external validity. The cross-sectional research design and the use of purposive sampling makes it difficult to draw causal inferences from the data.

Future work should test the boundary conditions of gendered wording effects and determine the smallest amount of gendered wording necessary to affect responses. The use of semi-structured interviews was justified here, but the sample of interviews could—in the future—be larger and include more diverse respondents. In addition, the data from the interviews could be subjected to inductive thematic qualitative analysis to identify themes that were not identified via the deductive directed qualitative content analysis that we applied to Study 2.

Future research should focus on examining the effects of gendered wording in other English-speaking countries, and countries outside of the English-speaking world. Content analysis and subsequent examination of currently used health promotion literature should be carried out to determine the extent of gendered wording usage and its perceived effectiveness. Similarly, content analysis of advertisements for socially important services (such as financial services) should be conducted and their effect on consumers evaluated. We also suggest that examining the effect of gendered wording on audiences representing different ethnic backgrounds is an important next step for this avenue of research.

The leaflets were not presented in the competitive environment of other persuasive materials. There may have been more time spent on the stimuli in this study in comparison to a real scenario (e.g. flipping through a magazine or reading a leaflet handed out on campus or at work, or looking at a website). Therefore, future studies should consider the possibility of testing the effect of wording in a more realistic environment. Given that social media communication is now often used, it would be important to test such effects when gendered content is presented by social media influencers [103]. As smart phone apps are often recommended for tracking and motivating physical exercise, it would be worthwhile to examine how wording of the text in smart phone apps influences usage of the app amongst men and women [104]. In addition, this study focused on featuring an expert (doctor), but the influence of the professional status of the endorser should also be examined [105]. The gender role identity of the endorser should also be manipulated and examined, in addition to the gender role identity of respondents, as well as the perceived fit/similarity of the endorser to the respondent. Finally, new social roles and self-identities are evolving in society, evident through studies in the transgender communities. This study could be replicated in transgender communities to test leaflet preference according to DGRI and gender identification.

## Conclusion

The purpose of the three studies was to examine the effect of gendered wording and the endorser’s gender in a health promotion message on attitude towards ad, ad credibility and behavioural intentions amongst male and female respondents in the UK. The findings suggest that gender role identity is an important variable that marketers should consider when targeting males, but not females. Specifically, masculine males should not be targeted with communal wording, as it is likely it will lead to negative evaluations. Females and feminine men, whilst they evaluated the gendered content of the leaflets differently, still evaluated them positively. The findings suggest women may conform less to traditional gender-role expectations than men, which may be due to advances to decrease the gender stereotyping of women [106–108].

## Supporting information

S1 Fig Male endorser and agentic wording leaflet. (TIF)

S2 Fig Female endorser and communal wording leaflet. (TIF)
