# Supplementary figures and images for: Masculine men do not like feminine wording: The effectiveness of gendered wording in health promotion leaflets in the UK
Source: PLoS One. 2022 Oct 27;17(10):e0273927. doi: 10.1371/journal.pone.0273927 (PMC9612536; doi:10.1371/journal.pone.0273927)

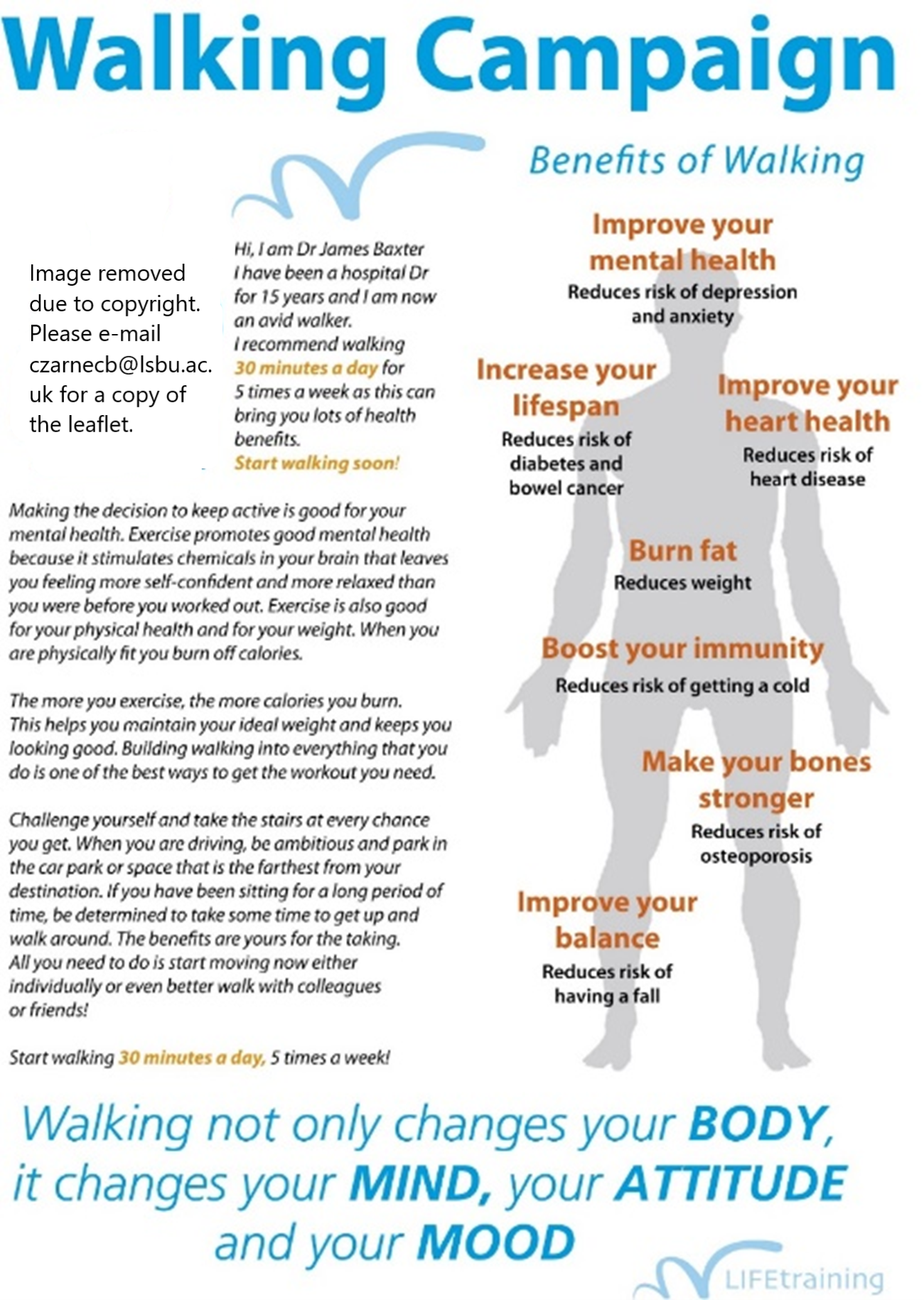

Supplement: S1 Fig — (TIF) [file pone.0273927.s001.tif]

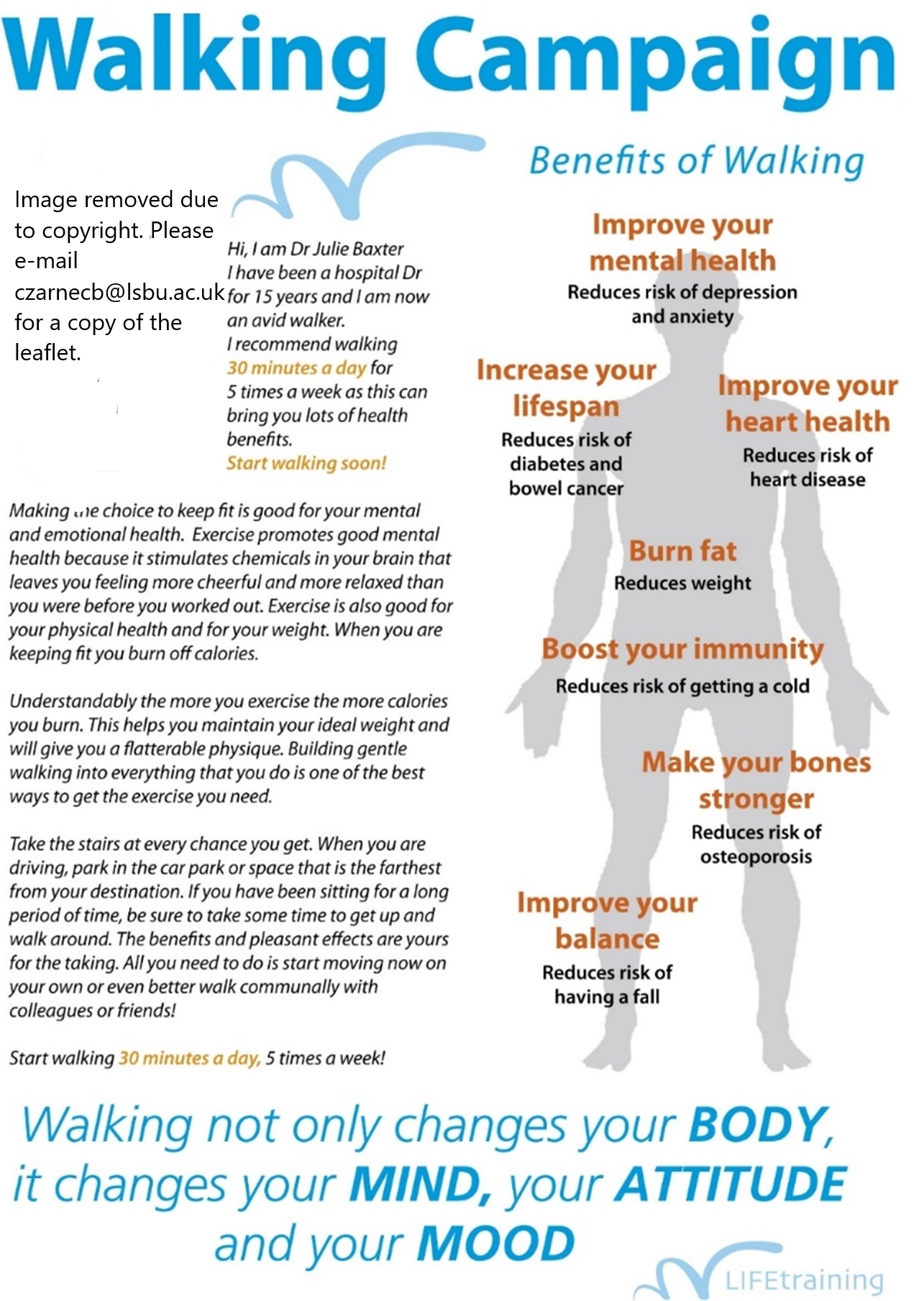

Supplement: S2 Fig — (TIF) [file pone.0273927.s002.tif]
